# Evolutionary history of the endangered fish *Zoogoneticus quitzeoensis *(Bean, 1898) (Cyprinodontiformes: Goodeidae) using a sequential approach to phylogeography based on mitochondrial and nuclear DNA data

**DOI:** 10.1186/1471-2148-8-161

**Published:** 2008-05-26

**Authors:** Omar Domínguez-Domínguez, Fernando Alda, Gerardo Pérez-Ponce de León, José Luis García-Garitagoitia, Ignacio Doadrio

**Affiliations:** 1 Posgrado en Ciencias del Mar y Limnología, ICMyL, Universidad Nacional Autónoma de México, C. P. 04510, D. F. México, México; 2 Laboratorio de Biología Acuática, Facultad de Biología, Universidad Michoacana de San Nicolás de Hidalgo, Morelia, Michoacán, México; 3 Departamento de Biodiversidad y Biología Evolutiva, Museo Nacional de Ciencias Naturales, CSIC, José Gutiérrez Abascal, 2, 28006 Madrid, España.; 4 Instituto de Biología, Universidad Nacional Autónoma de México, Ap. Postal 70-153. C.P. 04510 México D.F., México.

## Abstract

**Background:**

Tectonic, volcanic and climatic events that produce changes in hydrographic systems are the main causes of diversification and speciation of freshwater fishes. Elucidate the evolutionary history of freshwater fishes permits to infer theories on the biotic and geological evolution of a region, which can further be applied to understand processes of population divergence, speciation and for conservation purposes. The freshwater ecosystems in Central Mexico are characterized by their genesis dynamism, destruction, and compartmentalization induced by intense geologic activity and climatic changes since the early Miocene. The endangered goodeid *Zoogoneticus quitzeoensis *is widely distributed across Central México, thus making it a good model for phylogeographic analyses in this area.

**Results:**

We addressed the phylogeography, evolutionary history and genetic structure of populations of *Z. quitzeoensis *through a sequential approach, based on both microsatellite and mitochondrial cytochrome *b *sequences. Most haplotypes were private to particular locations. All the populations analysed showed a remarkable number of haplotypes. The level of gene diversity within populations was $\bar{H}$_*d *_= 0.987 (0.714 – 1.00). However, in general the nucleotide diversity was low, π = 0.0173 (0.0015 – 0.0049). Significant genetic structure was found among populations at the mitochondrial and nuclear level (Φ_ST _= 0.836 and *F*_*ST *_= 0.262, respectively). We distinguished two well-defined mitochondrial lineages that were separated *ca*. 3.3 million years ago (Mya). The time since expansion was *ca*. 1.5 × 10^6 ^years ago for Lineage I and *ca*. 860,000 years ago for Lineage II. Also, genetic patterns of differentiation, between and within lineages, are described at different historical timescales.

**Conclusion:**

Our mtDNA data indicates that the evolution of the different genetic groups is more related to ancient geological and climatic events (Middle Pliocene, *ca*. 3.3 Mya) than to the current hydrographic configuration of the basins. In general, mitochondrial and nuclear data supported the same relationships between populations, with the exception of some reduced populations in highly polluted basins (Lower Lerma River), where the effects of genetic drift are suggested by the different analyses at the nuclear and mitochondrial level. Further, our findings are of special interest for the conservation of this endangered species.

## Background

Primary freshwater fishes are strictly confined to freshwater basins, limiting their dispersal capacity. The evolution and dispersal of primary freshwater fishes are closely tied to the palaeogeography and history of connections, captures or separation of the water bodies they inhabit [1]. Accordingly, tectonic, volcanic and climatic events that produce changes in hydrographic systems are the main causes of diversification and speciation of freshwater fishes [2]. As these events reflect the geological development of landscapes, the phylogeographic studies of freshwater fishes permit to infer the biotic and geological evolution of a region [3].

The freshwater ecosystems of Central Mexico are characterized by their genesis dynamism, destruction, and compartmentalization induced by intense tectonic and volcanic activity. Its major physiographic feature is the Mesa Central, a large and isolated tropical highland, which includes the geological active Transmexican Volcanic Belt (TMVB), defined as the southern limit of the massive uplifted and as the transition area between the Nearctic and Neotropical provinces [4]. The tectonic activity of the Mesa Central started in the Miocene and reached its climax during the Pliocene-Pleistocene and has continued intermittently to the present, mainly in the TMVB region [5]. This intense geologic activity has generated a complex hydrologic system, which is the promoter of continuous processes of dispersion and vicariance. It has been suggested as the main cause for the high freshwater fish species richness (around 100 species) and unusual high levels of endemicity (around 70%) of the Mesa Central. Thus, this region is an interesting model for the understanding of the evolution and development of the biotic components of complex areas [6].

Many studies have discussed the biogeography of the Mesa Central, and have described the vicariant events that have resulted in subsequent differentiation in beetles [7], salamanders [8], toads [9], fishes [10] and mammals [11]. Most of the works centred in central Mexico have involved terrestrial taxa, but studies dealing with freshwater taxa are scarce [12]. More specifically, in the last years, the historical biogeography of the ichthyofauna of the Mesa Central of Mexico has been studied based on historical and descriptive methods of analysis [6,13-15]. These studies corroborated the pioneer works of several authors, who described general patterns of distribution of the freshwater fish fauna of the region, using occurrence data and detailed morphological comparisons (e.g. [16-18]). These contributions discussed diverse hypotheses, such as repeated events of connection and isolation of water bodies, river piracy, centers of origin, ancestral isolations between populations, and the effect of Pleistocene glaciations. However, these hypotheses have been widely debated and poorly understood [6,14,19].

Recent molecular studies have demonstrated the genetic signatures these volcanic, tectonic, and climatic events have left in some freshwater fish species of the Mesa Central, such as the Poecilids [10], Cyprinids [20] and Goodeines [21]. These studies have investigated the phylogenetic relationships at higher taxonomic levels and mainly evoke processes of isolation and vicariance. Nevertheless, to date, no specific evolutionary scenario of any freshwater organism has been proposed in the context of a phylogeographical approach with reference to climatic and geological events.

Within the endemic freshwater fish fauna of the Mesa Central, the Goodeinae is one of the most diverse groups (around 41 species), characterized by its particular life history, including internal fertilization, matrotrophy and viviparity, and a high degree of genetic divergence [15,21,22]. Within the Goodeinae, the genus *Zoogoneticus *is represented by two species, *Z. tequila *(Webb and Miller, 1998) and *Z. quitzeoensis *(Bean, 1898). The former is a microendemic species of the upper Ameca River basin, while the latter is widely distributed across the hydrological basins that drain the TMVB. Previous works have demonstrated that the populations of *Z. quitzeoensis *from Cuitzeo and Zacapu are significantly divergent when compared with populations from Lower Lerma [23] and Ameca basins [21]. However, no information about its evolutionary and demographic history has been yet provided. Further, the distribution range of this genus has been dramatically reduced due to habitat fragmentation and anthropogenic perturbations [24-26]. Because of these, *Zoogoneticus quitzeoensis *is considered an endangered species by the Mexican Official Norm of Ecology, and its sister species, *Z. tequila*, is now reported as extinct in the wild [27]. Thus, *Z. quitzeoensis *provides an interesting case-study for examining various features of the evolutionary and demographic history of the geologically active TMVB and its biota. Also, it can serve as a model to understand the processes and events that rule the biodiversity assemblages of the area and to promote its conservation.

In this sense, phylogeographical approaches have generally served to establish patterns of evolutionary history in distinct geographical populations. However, they have also been successfully used to infer historical demographic processes such as gene flow, effective population sizes or evolutionary trajectories [28]. Elucidating the evolutionary history of a species is important to understand population divergence and speciation and to provide more specific and accurate information of the processes and events that influence the evolutionary and demographic history of a region and its biota. Further, this information can be applied to conservation biology, as historical contingencies have been largely responsible for creating important genetic subdivisions in most extant taxa [29].

Mitochondrial DNA is preferentially and commonly used in most phylogeographic studies [30], although markers showing a faster evolution rate can uncover patterns on a more recent temporal scale [31]. Thus, the combined use of mtDNA and microsatellites has proved to be particularly effective for exploring both contemporary and historical events [32]. In this way, the sequential approach to phylogeography is recommended, as it examines both haplotype relatedness and demographic history [29,33]. This approach supports the idea that there is not one single and most powerful or informative analysis, but a combination of them [33]. Hence, the use of the sequential approach in phylogeography and different molecular markers, gives the opportunity to elucidate not only the spatial and temporal distribution of genealogical lineages, but the evolutionary and demographic history at different timescales.

Herein we describe the phylogeography, evolutionary and demographic history of *Zoogoneticus quitzeoensis *across its whole distribution range. Based on our results, we then infer the historical biogeographical scenario of its populations and propose future strategies for its conservation.

## Results

### Sequence variation and phylogenetic reconstruction

**Figure 1:**
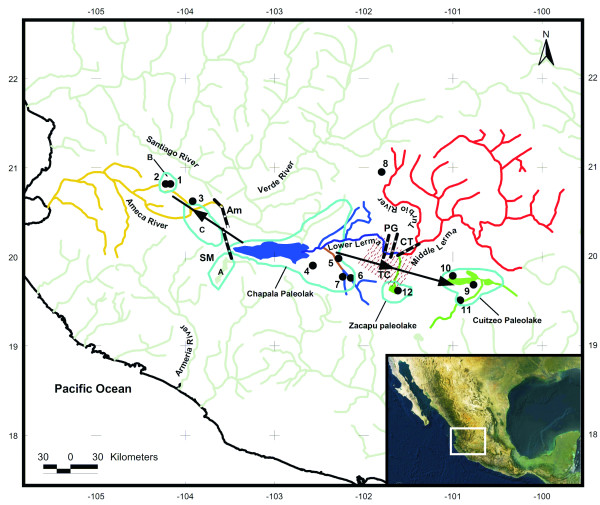
**Sampling sites in Central Mexico from which the *Zoogoneticus quitzeoensis *specimens were obtained**. 1) Magdalena, 2) El Moloya, 3) Los Veneros, 4) La Alberca, 5) La Platanera, 6) La Luz, 7) Orandino, 8) San Francisco del Rincón, 9) Belisario, 10) San Cristóbal, 11) La Mintzita, 12) Zacapu. Light blue outlines represent the areas of the paleolakes that developed in Central Mexico during the Miocene-Pleistocene: A) Sayula, B) Magdalena, C) Zacoalco-Ameca. Arrows represent proposed routes of colonisations. Black dotted lines represent geologic faults and grabens: Am: Ameca Fault, SM: San Marcos Fault, PG: Penjamillo Graben, CT: Chapala-Tula Fault, TC and dotted area: Corredor Tarasco volcanic field. The colours in the water bodies represent clades defined in Figures 2 and 3.

By sequencing 1140 bp of the entire mitochondrial cytochrome *b *gene in 80 individual *Z. quitzeoensis *specimens from 12 populations (Figure 1 and Additional file 1), 65 haplotypes were detected (Table 1). Fifty four sites were variable (4.73%), 37 were non-synonymous, and 17 were parsimony informative. As expected for a protein-coding gene, third codon positions were the most variable (10) followed by first (5) and second (2). The average ratio of non-synonymous/synonymous substitutions was *d*_*N*_*/d*_*S *_= 0.45 (CI: 0.35–0.56) and no evidence of positively selected sites was detected with any of the two methods used. Full details of substitution parameters and evolutionary models are given in Additional file 2.

**Table 1:** Measures of mitochondrial DNA diversity observed for the two lineages and other clades identified in this study.

Biogeographic region	Population	*N*	*Hn*	*S*	*H*_*d *_± SD	π ± SD	*k*
Lineage I		45	40	76	0.991 ± 0.008	0.0067 ± 0.0036	7.63 ± 3.73
Ameca River	Magdalena	6	5	6	0.933 ± 0.122	0.0017 ± 0.0013	2.00 ± 1.30
	Moloya	5	5	6	1.0 ± 0.126	0.0024 ± 0.0018	2.8 ± 1.77
	Veneros	9	9	19	1.0 ± 0.0524	0.0049 ± 0.0029	5.64 ± 2.98
	All populations	20	18	31	0.984 ± 0.018	0.0032 ± 0.0028	3.64 ± 1.39
Chapala Lake	La Alberca	7	7	16	1.00 ± 0.076	0.0044 ± 0.0028	4.97 ± 2.75
Lower Lerma River	La Platanera	5	5	8	1.00 ± 0.126	0.0028 ± 0.0020	3.21 ± 1.98
	La Luz	7	4	6	0.714 ± 0.181	0.0015 ± 0.0011	1.71 ± 1.13
	Orandino	6	6	13	1.00 ± 0.0962	0.0039 ± 0.0026	4.55 ± 2.60
	All populations	18	15	29	0.961 ± 0.039	0.0044 ± 0.0028	5.07 ± 1.37

Lineage II		35	25	54	0.946 ± 0.025	0.0061 ± 0.0033	6.97 ± 3.46
Middle Lerma River	San Francisco del Rincón	7	5	10	0.857 ± 0.137	0.0028 ± 0.0019	3.25 ± 1.90
Cuitzeo Lake	Belisario	6	6	13	0.714 ± 0.181	0.0049 ± 0.0032	5.69 ± 3.18
	San Cristóbal	7	4	9	1.00 ± 0.0764	0.0022 ± 0.0015	2.58 ± 1.56
	La Mintzita	6	5	9	0.936 ± 0.122	0.0032 ± 0.0022	3.75 ± 2.19
	All populations	19	14	27	0.936 ± 0.047	0.0039 ± 0.0024	4.52 ± 1.654
Zacapu Lake	Zacapu	9	7	9	0.917 ± 0.092	0.0017 ± 0.0012	2.00 ± 1.24

	Total	80	65	139	0.987 ± 0.007	0.0172 ± 0.0067	19.79 ± 1.96

N = sample size, *Hn *= number of haplotypes, *S *= number of polymorphic sites, *H*_*d *_= gene diversity, π = nucleotide diversity (Nei 1987), *k *= mean pairwise nucleotide diversity (Tajima 1993)

**Figure 2:**
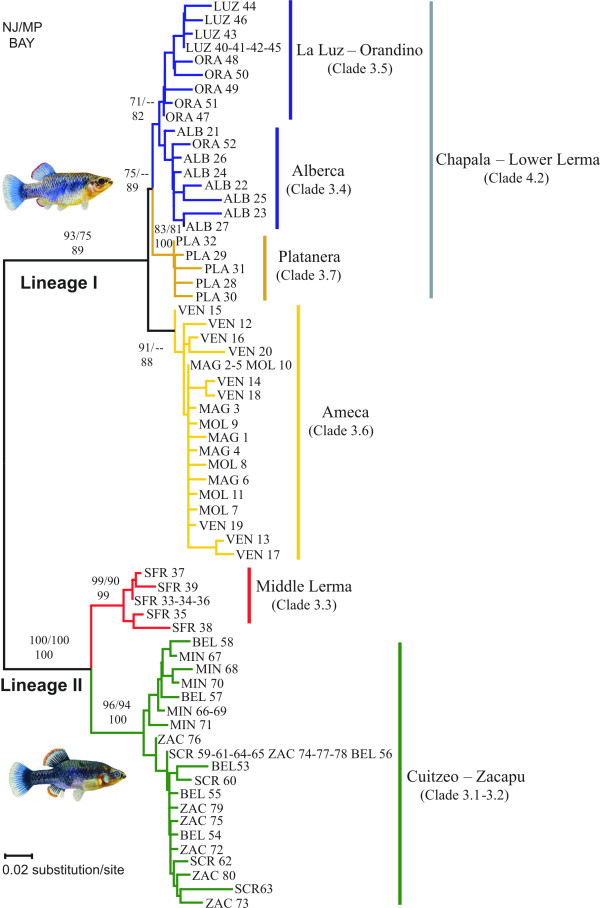
**NJ tree for the mtDNA haplotypes of the *Zoogoneticus quitzeoensis *populations examined in this study**. Bootstrap support of > 70% in NJ (top left) and MP (top right) and posterior probabilities (numbers below) for BI are given for the relevant nodes. The sister species, *Zoogoneticus tequila*, was used as outgroup (not shown).

Weighted parsimony analyses generated 95 equally parsimonious trees (length = 272, CI = 0.871, RI = 0.967). The three methods (NJ, MP and Bayesian) produced largely congruent tree topologies, with proportionally similar bootstrap and posterior probabilities supporting major lineage and population divergences (Figure 2).

Phylogenetic analyses identified two distinct lineages (Figure 2). Lineage I comprised 40 haplotypes distributed in the Lower Lerma and Ameca basins and in Lake Chapala, while Lineage II was composed of 25 haplotypes from the Middle Lerma Basin and from the Cuitzeo and Zacapu Lakes (Figure 2). Mean maximum likelihood and uncorrected *p *distance between the two lineages were $\bar{\text{D}}$_*ML *_= 3.05% ± 0.23 and $\bar{\text{D}}$_*p *_= 2.81% ± 0.21. Both lineages showed a well-defined internal structure (Table 2).

**Table 2:** Maximum likelihood and uncorrected *p *distances between clades and subclades of *Z. quitzeoensis*

	LUZ-ORA	ALB	PLA	AME	SFR	CUI-ZAC
LUZ-ORA	(0.30/0.31)	0.54	0.61	0.93	2.59	2.94
ALB	0.53	(0.43/0.43)	0.69	0.96	2.64	2.90
PLA	0.62	0.71	(0.25/0.25)	0.91	2.59	3.09
AME	0.90	0.99	0.89	(0.34/0.35)	2.67	2.93
SFR	2.79	2.85	2.79	2.88	(0.28/0.29)	1.21
CUI-ZAC	2.72	3.15	2.85	3.19	1.17	(0.37/0.38)

Above diagonal: uncorrected *p *distances; below diagonal: maximum likelihood distances (TIM+G); diagonal: within clade mean (%) distance (*p*/ML). LUZ: La Luz, ORA: Orandino, ALB: La Alberca, PLA: La Platanera, AME: Ameca, SFR: San Francisco del Rincón, CUI: Cuitzeo, ZAC: Zacapu.

Within Lineage I, we found two main clades. One clade (Ameca Clade) included 18 haplotypes (*Hn*) from the 3 sampled sites in the Ameca River Basin (Los Veneros, Magdalena and Moloya), and the other clade (Chapala-Lower Lerma Clade: *Hn *= 22) contained individuals from the localities sampled in the Lower Lerma Basin and Lake Chapala. Distances between Ameca and Chapala-Lower Lerma clades were $\bar{\text{D}}$_*ML *_= 0.93% ± 0.2 and $\bar{\text{D}}$_*p *_= 0.92% ± 0.2.

In Lineage II, the first clade comprises the Lake Cuitzeo and Zacapu Lake Basin populations (Cuitzeo-Zacapu Clade: *Hn *= 20), and the second one, the San Francisco del Rincón population (Middle Lerma Clade: *Hn *= 5). The $\bar{\text{D}}$_*ML *_between the two clades was 1.17% and $\bar{\text{D}}$_*p *_= 1.21 (Table 2).

### Nested clade analysis

**Figure 3:**
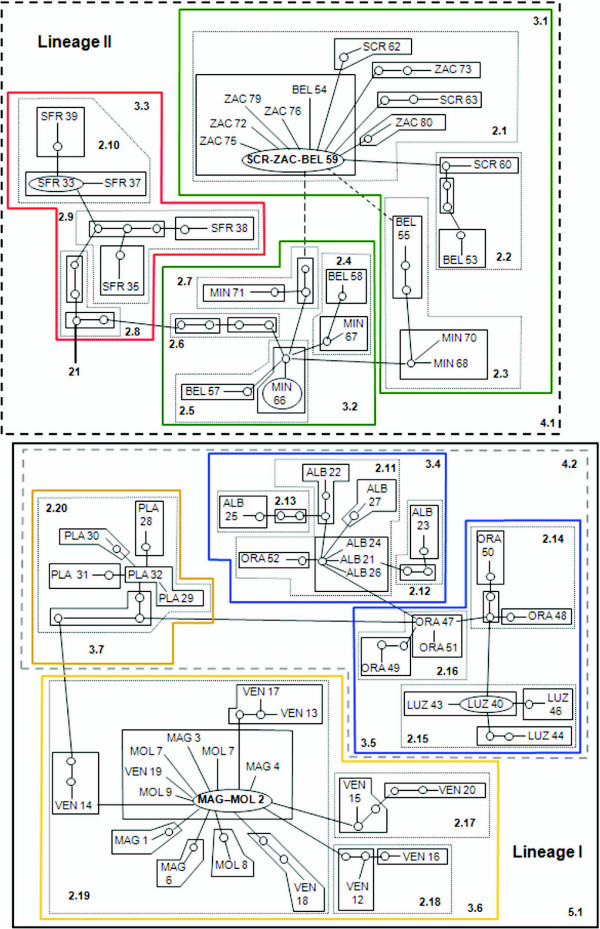
**Statistical parsimony (95%) network of 68 mitochondrial DNA haplotypes identified for *Zoogoneticus quitzeoensis***. The network was grouped into nesting clades. Each line in the network represents a single nucleotide substitution. Small circles indicate undetected intermediate haplotype states. Ovals represent haplotypes with more than one specimen. The number 21 represents the number of mutational steps between the two upper clades.

The statistical parsimony haplotype network for *Z. quitzeoensis *indicated a similar pattern to that revealed by the phylogenetic analysis and showed the existence of two well-defined evolutionary lineages (Figure 3). The number of mutational steps (21) between lineages exceeded the maximum number of mutational connections justified by the 95% parsimony criterion (14 mutational steps). Thus, each lineage was treated independently in subsequent analyses.

The null hypothesis of the nested contingency analysis of no association between haplotype positions in the cladogram and their geographical locations was rejected in six of the tests performed (*P *< 0.05) (Figure 3 and Additional file 3).

Allopatric fragmentation could have rendered the geographical pattern of the full cladogram of *Z. quitzeoensis*. In Lineage I, the five-step clade showed significant values; there was insufficient evidence, however, to discriminate between range expansion, colonization and restricted dispersal or gene flow. Within this lineage, long distance colonization and/or past fragmentation was inferred for Clade 3.6 (Clade Ameca) and long distance colonization associated with subsequent fragmentation followed by range expansion for clades 3.5 (Clade La Luz-Orandino) and 4.2 (Clade Chapala-Lower Lerma) [34].

In Lineage II, the inference key [34] suggested long distance colonization and/or past fragmentation for Clade 4.1 (Lineage II).

### Genetic structure

The mtDNA Φ_ST _values obtained for the comparisons of all the populations ranged from 0.006 to 0.940 (Table 3). Highest significant values were obtained for most comparisons involving the Zacapu population (Φ_ST _= 0.518 – 0.940), except for comparisons with two populations from Lake Cuitzeo (Belisario and San Cristóbal). Significant Φ_ST _values were also obtained for all the comparisons of Los Veneros with the rest of the populations, except for those involving populations within the same basin (Magdalena and Moloya). The only significant Φ_ST _values between samples within the same basin were found in the Lower Lerma River, except for Orandino and La Platanera (Table 3).

**Table 3:** Estimate pairwise comparisons of cytochrome b sequences (mtDNA) above the diagonal (Φ_ST_) and for five microsatellite loci below the diagonal (F_ST_) for the *Zoogoneticus quitzeoensis *populations.

	MAG	MOL	VEN	ALB	PLA	LUZ	ORA	SFR	BEL	SCR	MIN	ZAC
MAG	-	0.006	0.020	**0.654**	0.736	0.814	0.652	0.912	0.888	0.932	0.914	**0.940**
MOL	0.025	-	0.012	0.624	0.702	0.790	0.618	0.901	0.874	0.923	0.902	**0.933**
VEN	-	-	-	**0.545**	**0.574**	**0.656**	**0.519**	**0.855**	**0.839**	**0.877**	**0.857**	**0.889**
ALB	-	-	-	-	0.488	0.483	0.141	0.868	**0.847**	**0.890**	**0.870**	**0.903**
PLA	**0.337**	**0.316**	-	-	-	**0.686**	0.437	**0.894**	0.865	0.916	0.894	**0.927**
LUZ	**0.363**	**0.354**	-	-	**0.408**	-	**0.325**	**0.347**	0.889	0.932	0.915	**0.939**
ORA	**0.132**	**0.111**	-	-	**0.224**	**0.271**	-	**0.091**	0.843	**0.893**	0.870	**0.906**
SFR	**0.315**	**0.330**	-	-	**0.352**	**0.455**	**0.258**	-	**0.682**	**0.785**	**0.738**	**0.808**
BEL	**0.321**	**0.300**	-	-	**0.368**	**0.345**	**0.236**	**0.179**	-	0.076	0.137	0.115
SCR	**0.283**	**0.268**	-	-	**0.371**	**0.380**	**0.243**	**0.132**	0.034	-	0.477	0.005
MIN	**0.278**	**0.258**	-	-	**0.377**	**0.406**	**0.249**	**0.170**	**0.059**	**0.046**	-	**0.518**
ZAC	**0.343**	**0.309**	-	-	**0.397**	**0.355**	**0.231**	**0.209**	**0.041**	**0.107**	**0.082**	-

Significant values after Bonferroni correction are shown in bold.

MAG, Magdalena; MOL, Moloya; VEN, Veneros; ALB, Alberca; PLA, Platanera, LUZ, La Luz; ORA, Orandino; SFR, San Francisco del Rincón; BEL, Belisario; SCR, San Cristobal; MIN, Mintzita; ZAC, Zacapu.

Pairwise *F*_*ST *_values based on microsatellite data were significant for most of the comparisons, except for Moloya-Magdalena in the Ameca basin and for Belisario-San Cristóbal in the Cuitzeo Lake basin (Table 3). Highest *F*_*ST *_values were observed for the comparisons between La Luz and San Francisco del Rincón, and La Platanera with La Mintzita (*F*_*ST *_= 0.455, 0.408 and 0.406 respectively). Although the La Luz and La Platanera populations occur in the same basin (Lower Lerma), their *F*_*ST *_values were among the highest. Notwithstanding, the other population inhabiting the Lower Lerma Basin (i.e. Orandino) showed significant yet much lower *F*_*ST *_values when compared with La Luz and La Platanera (*F*_*ST *_= 0.271 and 0.224 respectively).

The AMOVA performed for the mitochondrial and nuclear data, revealed significant structure among populations (Φ_ST _= 0.836, *P *< 0.001 and *F*_*ST *_= 0.262, *P *< 0.001 respectively). For the subsequent analyses, populations were grouped in different hierarchical arrangements according to previous information and to uncover groupings obtained in the previous analyses (e. g. phylogenetic analysis and NCA) and by their biogeographical arrangement (Table 4). Significant values were obtained when the biogeographic arrangement of basins [6] was considered (Φ_CT _= 0.787, *P *< 0.001, *F*_*CT *_= 0.107, *P *< 0.05). When the basins where each lineage was found were considered separately, only Lineage I showed significant structure for mtDNA (Table 5). When two gene pools corresponding to the two lineages found were considered, 74.8% (*P *< 0.001) of the total variance was explained as differences among groups for mtDNA and 11.18% in the case of microsatellite data (*P *< 0.01). Division of the populations into the groups suggested by the phylogenetic and NCA analysis maximised among-group variance for the mtDNA data (Φ_CT _= 0.823, *P *< 0.001). Thus, the structure previously obtained in the phylogenetic and NCA was statistically supported for the mtDNA, but not for the microsatellite data.

**Table 4:** Hierarchical analysis of molecular variance based on mtDNA haplotypes and microsatellite allele frequencies among *Z. quitzeoensis *populations.

Groups	*F*_*ST*_	*F*_*CT*_	*F*_*SC*_	% Among groups	% Within groups	*P*
mtDNA						
One gene pool (Populations)	0.836	-	-	83.63	16.37	< 0.001
Biogeography (Ameca)(Chapala)(Lower Lerma)(Middle Lerma-SFR)(Cuitzeo)(Zacapu)	0.849	0.787	0.292	78.7	15.08	< 0.001
Lineage I Biogeography (Ameca) (Chapala) (Lower Lerma)	0.613	0.462	0.280	46.23	38.69	< 0.05
Lineage II Biogeography (Middle Lerma- SFR)(Cuitzeo)(Zacapu)	0.643	0.487	0.305	48.71	35.65	ns
Two gene pools (Lineage I)(Lineage II)	0.893	0.748	0.574	74.8	10.72	< 0.001
Phylogenetic arrangement (Moloya-Magdalena-Veneros)(Belisario-Zacapu-San Cristóbal-Mintzita)(San Francisco del Rincón)(La Platanera)(Orandino-La Luz-La Alberca)	0.832	0.823	0.168	84.32	14.71	< 0.001
nDNA						
One gene pool (Populations)	0.262	-	-	26.18	73.82	< 0.001
Biogeography (Chapala)(Bajo Lerma)(Middle Lerma-SFR)(Cuitzeo)(Zacapu)	0.275	0.107	0.275	10.76	72.47	< 0.05
Lineage I Biogeography (Ameca)(Chapala)(Bajo Lerma)	0.323	0.082	0.263	8.27	67.63	ns
Lineage II Biogeography (SFR)(Cuitzeo)(Zacapu)	0.135	0.094	0.046	9.37	86.49	ns
Two gene pools (Lineage I)(Lineage II)	0.298	0.112	0.210	11.18	70.14	< 0.01

**Table 5:** Estimates of demographic parameters and neutrality tests within the two species and main clades obtained.

	Clade	τ	*F*_*s*_	*D*	*H*_*ri*_
Lineage I	5.1	5.647	-38.98**	-2.03*	0.0049ns
	3.6 Ameca	5.08	-18.40**	-2.27*	0.0249ns
	4.2 Chapala-Lower Lerma	5.647	-15.71**	-1.97*	0.0161ns
Lineage II	4.1	3.09	-13.26**	-1.76*	0.0124ns
	3.1–3.2 Cuitzeo-Zacapu	2.83	-15.22**	-2.13*	0.0218ns

τ = age expansion parameter, *F*_*s *_= Fu's statistic, *D *= Tajima's *D *test, *H*_*ri *_= Harpending's raggedness index. * *p *< 0.05, ** *p *< 0.01, ns = not significant

**Figure 4:**
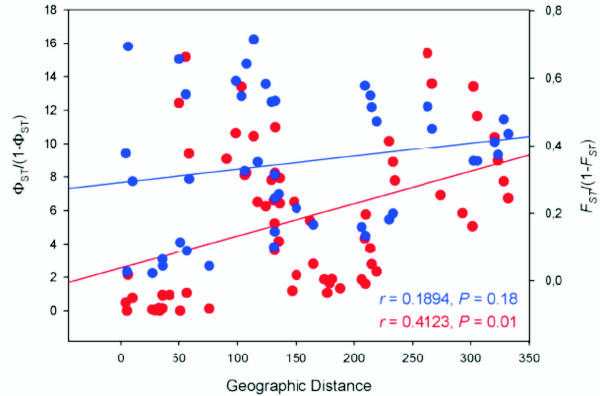
**Correlation between geographic distances and genetic distances among all populations of *Z. quitzeoensis***. Plot of geographic distances against the genetic distances for mtDNA (Φ_ST_/1-Φ_ST_) in red, and the genetic distances for microsatellite data (F_ST_/1-F_ST_) in blue. Mantel test's *r *and *P *values are shown.

The Mantel test revealed a significant correlation between geographical and mitochondrial genetic distances (*r *= 0.4123, *P *= 0.01). Otherwise, non significant correlation was found considering the genetic distances obtained using the microsatellite data (*r *= 0.1894, *P = *0.179) (Figure 4).

**Figure 5:**
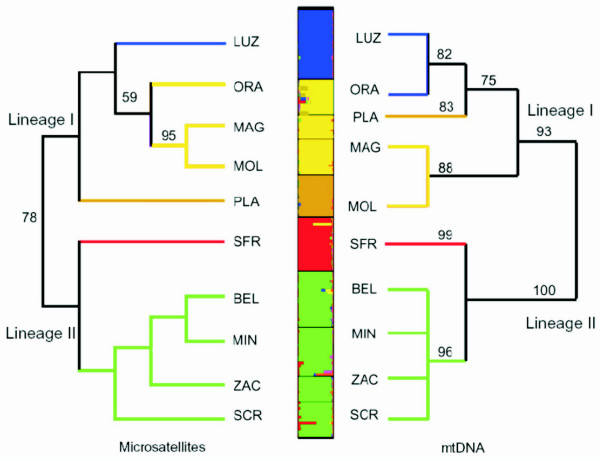
**Phylogenetic relationships of *Zoogoneticus quitzeoensis *populations**. Left: NJ tree based on distances calculated for the microsatellite loci. Right: Bayesian tree based on mtDNA data for the same populations used in the microsatellite analysis. Centre: Bar plot obtained using STRUCTURE for the most likely number of clusters *K *= 5. LUZ: La Luz, ORA: Orandino, MAG: Magdalena, MOL: Moloya, PLA: La Platanera, SFR: San Francisco del Rincón, BEL: Belisario, MIN: Mintzita, ZAC: Zacapu, SCR: San Cristóbal. Colours represent clades defined in Figures 2 and 3, except Orandino, in yellow, which appears differentiated from La Luz and clustered with El Moloya and Magdalena populations at the nuclear level.

The Bayesian structure analysis for the microsatellite data clearly revealed a genetic structure among the specimens analysed. According to the maximum likelihood value, we estimated a number of genetic clusters of *K *= 6. Estimates of lnPr(*X*|*K*) increased rapidly between *K *= 1 and *K *≤ 6, whereas beyond *K *> 6 lnPr(*X*|*K*) started to oscillate. But when we considered *Δ K*, we obtained clear peak at *K *= 5 (Figure 5). Conversely, the highest global *F*_*ST *_value was found for *K *= 6, indicating that these clusters explained the maximum level of structure in our sample (Additional file 4). However, for the following discussion we adopted the more conservative measure of *K = *5, obtained with the correction of Evanno *et al*. [35]. All populations were assigned with high probability (*Q *= 0.844 – 0.962) to their inferred cluster.

The results derived from the microsatellite NJ tree were highly congruent with those from the Bayesian clustering analysis and most of the phylogenetic mtDNA tree, but with some differences within Linage I (Figure 5). Two well-supported lineages were obtained. In Lineage II, all samples from the Cuitzeo and Zacapu basins grouped together, and San Francisco del Rincón appeared differentiated from these. Otherwise, Lineage I showed a higher degree of structure, with its populations distributed in three different clusters (Figure 5). When considering *K *= 6, Orandino represented a separate cluster. Slightly different results, regarding the position of the Lower Lerma populations, were rendered by the two types of marker for population relationships (Figure 5). The population assignment test correctly assigned 80.59% of the individuals to their original population. While for the Lineage I populations, 91.5% correct assignments were obtained, in Lineage II, gene flow was found among all populations (72% of the individuals were assigned correctly) except for the site San Francisco del Rincón, whose individuals were all unequivocally assigned.

### Mitochondrial DNA variation and demographic patterns

Overall gene diversity was *H*_*d *_= 0.987 and nucleotide diversity π = 0.0172. Most haplotypes were found at single sites. Only two haplotypes were shared among individuals from different localities. One of them was also the most common haplotype overall, found in 8 individuals from the sites Zacapu, San Cristóbal and Belisario (ZAC-SCR-BEL 59), and the other one was found in 3 individuals from Magdalena and Moloya (MAG-MOL 2) (Figures 2 and 3). The rest of the haplotypes were shared among individuals within the same localities.

**Figure 6:**
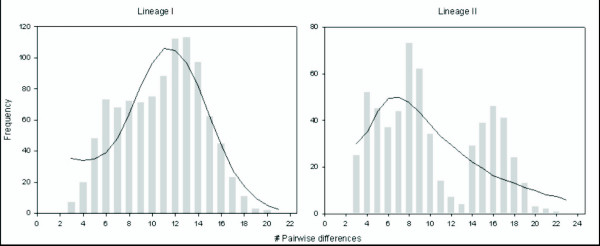
**Mismatch distribution for the two main lineages obtained in the cladogram and NCA**. Grey bars indicate the observed values and black lines show the expected distribution under the sudden expansion model.

All the populations analysed showed a remarkable number of haplotypes (Table 1). The level of gene diversity within populations was $\bar{H}$_*d *_= 0.987 (0.714 – 1.00). However, the nucleotide diversity exhibited by most of the tested populations (π = 0.0015 – 0.0049) was low. Mean pairwise nucleotide diversity (*k*) ranged from 1.71 to 5.69 within populations (average number of nucleotide differences for the whole sample *k *= 19.79; Table 1). The mismatch distribution (MMD) for all the data set was bimodal (not shown), one of the peaks represents the differences between lineages, and the other the differences among individuals within lineages. The two lineages and the different clades obtained in the previous analyses were tested independently. The MMD for Lineage I was unimodal and bimodal for Lineage II (Figure 6), which is expected when populations are geographically subdivided [36]. In both cases, raggedness indices (*r*) were not significant, thus not rejecting the null hypothesis of stationarity. Conversely, Tajima's *D*-statistic and Fu's statistic (*F*_*s*_) were significantly negative and supported the expansion model for the different groups tested (Table 5). Both tests show a significant excess of the number of segregating sites and singletons compared to the average pairwise sequence divergence. These tests indicate that the different groups analyzed are in mutation-migration-drift genetic disequilibrium with respect to mtDNA alleles. The different results obtained by these three demographic tests might be due to the low power of mismatch distribution based tests (e.g. raggedness), compared with methods based on the mutation frequency (e.g. Tajima's *D*) or haplotype distribution (e.g. Fu's *F*). In a variety of cases and for large samples sizes, Fu's *F *has been proved to be the most powerful test to detect population growth [37]. Values of τ differed between lineages and among clades. For Lineage I, the mean of the mismatch distribution was τ = 5.647 and the estimated time since population growth was 1.5 × 10^6 ^years before present. Within Lineage I, the estimated time since population expansion for the two clades obtained (Ameca and Chapala-Lower Lerma) were *ca*. 1.4 × 10^6 ^(τ = 5.08) and *ca*. 1.08 × 10^6 ^(τ = 5.647), respectively. In Lineage II, we estimated *ca*. 860,000 years since population expansion according to the value of τ = 3.09. For the clades within Lineage II, the estimates of time since population expansion were *ca*. 790,000 years for Cuitzeo-Zacapu (τ = 2.83) and *ca*. 550,000 years for San Francisco del Rincón (τ = 1.95).

## Discussion

### Phylogeography and evolutionary history

The trees obtained by the different methods (NJ, MP and BI) using both types of markers (cytochrome *b *and microsatellites) distinguished two independent lineages, supporting the conclusions of previous studies [21] in which two divergent groups were identified within *Z. quitzeoensis*. Lineage I inhabits areas west of the Middle Lerma, including the Lower Lerma, Ameca and the Chapala Lake basins. Lineage II occurs at sites east of the Angulo River and the Middle Lerma Basin, including the Cuitzeo and Zacapu lakes. Our NCA also supported this conclusion, indicating the two main lineages were not nested together; moreover, the 21 mutation steps between the two lineages exceeded the 95% parsimony limits (14 mutation steps). This result suggests allopatric fragmentation between the two clades.

The formation of the two lineages of *Z. quitzeoensis *was dated at *ca*. 3.3 Mya based on the molecular clock calibration of 0.9% divergence per million years [21]. These two lineages could be the result of a dispersal process of the ancestral population from the Lower Lerma-Chapala area to the Middle Lerma-Cuitzeo-Zacapu area (Figure 1), followed by an isolation event. This hypothesis is based on the facts that: Lineage I is the most widely distributed group (distributed in Chapala-Ameca-Lower Lerma drainage), and it shows, overall, a higher genetic diversity than Lineage II (*Hd *= 0.991, *S = *76 and *Hd *= 0.946, *S *= 54, respectively).

The dispersion event we refer to, from the Chapala-Lower Lerma to the Middle Lerma-Cuitzeo-Zacapu area, could have been promoted by a period of high precipitation and humidity occurred in Central Mexico in the early Pliocene (5.2 – 3.6 Mya). This could have caused an increase of the water bodies level in this area causing them to get in contact, as was previously proposed for other lakes in Central Mexico [38,39]. The subsequent isolation of the ancestor of the two lineages that induced allopatric fragmentation, could have been the result of the end of the humid period, and/or the formation of a biogeographical barrier promoted by the geologic activity of the Penjamillo Graben [40,41], the Chapala-Tula fault or the activity and formation of the Corredor Tarasco volcanic field, which commenced during the Late Miocene-Early Pliocene [40] (Figure 1). This climatic change and the high tectonic activity have been proposed as the causes for the isolation of the palaeolakes along the TMVB during the Late Miocene-Pliocene (Figure 1) [38].

Since recent geological times, some of the regions where these two lineages occur have been connected, and at present constitute one single hydrologic system (Lerma River). However, our findings of ancestral isolation between Lower Lerma-Chapala and Middle Lerma-Zacapu-Cuitzeo are supported by at least one pair of sister species with the same cladogenetic pattern, *Skiffia lermae*-*S. multipunctata*, dated around 3.2 Mya [21]. This indicates that the same biogeographic event could have promoted the isolation of the two divergent groups and consequently they could be considered as two ESUs [42,43]. Furthermore, considering the morphological differences between groups, and pending of more detailed morphometric studies, they could be considered as two species.

### Within lineage genetic structure and demography

The results of all our analyses revealed significant genetic structure and differentiation among populations between and within the two lineages, but with certain differences as indicated by the two types of molecular markers.

#### Lineage I: Ameca-Lower Lerma-Chapala area

The two main clades identified within Lineage I correspond to two different hydrologic systems. One clade appears in the Ameca river basin (Clade 3.6) and the other is distributed across the Lower Lerma-Chapala Lake area (Clade 4.2). When Lineage I was tested independently, the statistical associations in the nested contingency analysis were not able to discriminate between range expansion and colonization *versus *restricted dispersal and gene flow. However, the fact that haplotype LUZ-40 emerged as the most probable outgroup in the network, and the significant negative value of the Fu's *F*_*s*_, and Tajima's *D *statistics seem to better support the range expansion and colonization scenario. The haplotype arrangement found within the three populations sampled on the Ameca river basin also supports this scenario. Haplotypes from Los Veneros are placed in a basal position in the phylogenetic trees and appeared in all the 2-step clades found in the NCA (within 3.6 Clade). Such patterns could indicate that Los Veneros may represent the ancestral population of the Ameca Basin. The Los Veneros population is geographically close to the San Marcos-Atotonilco lakes (~19 Km), which formed part of the Chapala Palaeolake [44], but also it is close to the Zacoalco-Ameca paleolake. This supports the hypothesis that *Z. quitzeoensis *spread from the Chapala Palaeolake region to the Ameca River region via the Zacoalco-Ameca Paleolake (Figure 1). The expansion for the Ameca River populations was calculated in *ca*. 1.4 × 10^6 ^(τ = 5.08), in such case the dispersion event could have taken place at the beginning of the Pleistocene.

A former connection between the Chapala and Ameca basins have been previously proposed via the Atotonilco and San Marcos lakes [45] (Zacoalco-Ameca Paleolake area). Other authors have supported this connection based on the distributions of related species/population pairs of fish, as in *Poeciliopsis *[10], *Notropis *[46], *Chirostoma *[17], *Ictalurus *[19], *Yuriria *[47] and almost three events of Goodeines exchange [6,21].

Within Lineage I, another two clades showed a significant association in the geographic contingency test: Clade 4.2, in which specimens from the Chapala and Lower Lerma were included; and Clade 3.5, comprised of the geographically close populations of La Luz and Orandino that are ~4 km apart within the same basin. In both cases, long distance colonisation possibly accompanied by subsequent fragmentation or past fragmentation followed by range expansion, was inferred from the NCA. Moreover, the genetic distance between the population of La Platanera (clade 3.7) and the populations of La Luz and Orandino (clade 3.5), within the Lower Lerma region, was larger than the distance between the population of La Alberca (clade 3.4), in the Chapala Lake region, and the populations of Orandino and La Luz (Table 2). These results are in disagreement with a previous biogeographic hypothesis, where the Lower Lerma and Chapala Lake were considered as independent biogeographic entities [6]. The significant outcome of Clade 3.5 (populations within the Lower Lerma region) might also be due to the fact that NCA is likely to give false-positive results, and consequently detect a significant spatial structure, in populations that have suffered processes that affect local haplotype frequencies, such as bottlenecks [48]. This could be the case for La Luz and Orandino where the effects of genetic drift and low population size (e.g. low genetic diversity and significant inbreeding), caused by human activities (e.g. pollution, desiccation and isolation of the water bodies, introduction of exotic species) have been proved [49] and are congruent with the high genetic differentiation and significant inbreeding revealed by the microsatellite data (Table 3, Figure 5, Additional file 5).

The pronounced genetic structure among the contiguous sampling sites of Orandino, La Platanera, and La Luz was also found at the nuclear level (Table 3, Figure 5). Further, most of the differences between the mtDNA and nDNA analyses were found in relation to this area (Figure 5). Thus, all these results suggest that recent demographic events could have shaped, through genetic drift, the genetic structure of the populations in the Lower Lerma basin.

#### Lineage II: Middle Lerma-Cuitzeo-Zacapu area

In Lineage II, two well-differentiated groups were recovered by the mtDNA (i.e. Φ_ST_, NCA and phylogenetic trees) and microsatellite analyses (i.e. STRUCTURE, *F*_*ST *_and NJ tree). The first of these groups comprises the San Francisco del Rincón population and the second group includes the populations from Zacapu, La Mintzita, San Cristóbal and Belisario, the last three belonging to the Cuitzeo region. In the NCA, only Clade 4.1, which clustered together all the populations of this lineage, showed a significant geographical association, but insufficient to discriminate between long distance colonization and past fragmentation (Additional file 3).

According to our demographic results, both scenarios provided by the NCA inference key are candidates for the five populations distributed across three different basins (Additional file 3). However, certain features, for instance: the low genetic diversity in the San Francisco del Rincón population suggested by the mtDNA and confirmed by previous microsatellite studies [49] (Additional file 5), and that this population probably expanded more recently (*ca*. 540,000 years), could point to the long distance colonization of organisms from Zacapu-Cuitzeo and past fragmentation as the most plausible scenario. This assumption is supported by the neutrality tests of Fu's *F*_*s *_and Tajimas's *D *statistics.

All the populations within this lineage showed evidence of recent gene flow, except the one from San Francisco del Rincón. Our results also indicated non significant genetic differentiation for most of the pairwise comparisons, for mtDNA, among the four populations inhabiting the Zacapu and Cuitzeo basins. Haplotypes were shared by the two basins, a fact that disagrees with the hypothesis that Zacapu and Cuitzeo constitute two well-defined biogeographic entities [6]. Although the two basin populations are close (≈50 km), at present they are geographically separated by a mountain chain. The time since expansion for these populations was estimated at *ca*. 790,000 years ago. Our results support the idea of an ancient connection between Zacapu and Cuitzeo lakes, via a river located in the Chucandiro-Uaniqueo region, and further disrupted by the geologic and volcanic activity during the Plio-Pleistocene (≈1 Mya) [14]. This connection is also stated in previous phylogenetic studies that address population relationships between Zacapu and Cuitzeo based on species of the genus *Notropis *[20] and other Goodeinae species [21]. However, we found differences in the divergence times, suggesting that more than one event of connection and isolation between the Zacapu and Cuitzeo regions occurred in the last three million years.

### Discrepancies between nuclear and mitochondrial markers

Mitochondrial and nuclear markers reveal different parts of the evolutionary history of the organisms due to their different inheritance modes and mutation rates. Even though mitochondrial markers coalesce faster than nuclear markers due to the smaller effective size and the lack of recombination, microsatellites have a much faster mutation rate which makes them useful to detect more recent processes [50,51].

In general, the relationships inferred with the mitochondrial and microsatellite data set were congruent, but some differences were found. Whereas both markers retrieved the existence of two main clades, the relationships within them were not the same (Figure 5). Discrepancies were observed in Lineage I for the comparisons of the Chapala-Lower Lerma populations. In this case, both makers showed significant differences among geographically close populations. High and significant pairwise *F*_*ST *_values were obtained for most of the comparisons of populations from the Lower Lerma basin (Table 3), which also were assigned to different genetic clusters by the Bayesian clustering analysis and microsatellite NJ tree, but formed a single clade according to mtDNA (Figure 5). Some of these localities have recently suffered a severe reduction in the size of their water bodies, and become isolated with a consequent increase of the effects of genetic drift, resulting in genetic differentiation. Moreover, it has been suggested that the Lower Lerma populations suffered a more intense genetic drift than other populations within *Z. quitzeoensis *[49]. Similarly the non-significant correlation between geographic and genetic distances based on the microsatellite data might be explained by the stochastic processes that are recently shaping the genetic structure of *Z. quitzeoensis*, and consequently the high genetic differences between nearby localities.

The differences found in the genetic diversity values were much higher for the microsatellite than the mitochondrial data, which showed a high diversity for all populations (Table 1 and Additional file 5). The populations of Zacapu and San Francisco del Rincón, which showed allelic richness values of only 2.93 and 2.97, still retained haplotypic diversities of 0.917 and 0.857. Thus, eventhough samples sizes are, in some cases, low and unequal we might deduce that the loss of diversity is occurring at a different rate for the two types of markers [52]. Additionally, none of the Tajima's *D *values, either for single populations or lineages, were significantly positive, which would be indicative of a bottleneck [53], whereas seven out of the 10 populations analyzed showed a significant signature for a recent bottleneck, with at least one of the three microsatellite mutational models (Additional file 5).

### Implications for conservation

The importance of fish diversity in Central Mexico has been largely recognized [54] but very few efforts have been made to establish a basis for their conservation. Further, no studies have addressed the evolutionary processes underlying such diversity targeted at maintaining these processes. Our results, derived from both phylogeographic and population analyses, could prompt certain conservation management strategies. First, the two reciprocally monophyletic lineages of *Z. quitzeoensis*, support the idea that future conservation plans should be aimed at managing the populations of both lineages independently and, furthermore, be considered as two ESUs [42,43].

Moreover, within each lineage, we found genetic structure for the two markers, supporting previously identified Operational Conservation Units (OCU's) for *Zoogoneticus *[49]. When the species under study shows high genetic structure, ideally all the populations should be protected since they contain unique portions of the total variation of the species. This may increase the adaptation and the survival possibilities of the species as a whole.

## Conclusion

The present study is the first attempt to describe, on a fine scale, the evolutionary history of populations of a fish species in Central Mexico. Our results demonstrate the value of the use of mtDNA combined with nuclear microsatellite loci to detect genetic structure and to elucidate the evolutionary history of fish species. The methodology used integrates independent geological and genetic information to test for interactions between historical and contemporary factors in a highly structured endemic fish in Central Mexico.

Our results indicate that the evolutionary history and genetic structure among populations of this fish species is closely tied to geological and climatic events that promoted changes in the ancient drainages, since the Middle-Pliocene, rather than to the current configuration of those drainages. In addition to this, the results obtained and differences between molecular markers are an evidence of the effects of genetic drift over the genetic structure in some highly polluted aquatic environments.

The information provided by this type of study is essential for the conservation of highly genetically structured species and its phylogeographic hypotheses prompt comparable analyses of other codistributed fish species to test the scenarios proposed.

## Methods

### Specimen collection and DNA extraction

Fin clips were obtained from individuals of *Zoogoneticus quitzeoensis*. Specimens were collected from 12 sites distributed across six regions along the Mesa Central of Mexico (Figure 1 and Additional file 1), representing most of the distribution range of the species. The fishes were sampled using minnow traps, seine nets and by electrofishing. Tissue was preserved in 96% ethanol and most fishes were returned to the water unharmed. A few fish specimens were incorporated into the Goodeid Conservation Program of the Universidad Michoacana de San Nicolás de Hidalgo. Because of the endangered status of *Z. quitzeoensis *[27] and its scarcity at all the collection sites, sample sizes ranged from 7 to 20 individuals per population (Additional file 1). Similar sample sizes have been used successfully in similar phylogeograpic studies of freshwater fauna [55-61]. However, it has long been proved that phylogenetic and population genetic inferences are sensitive to the number of taxa included. The number of specimens and populations needed to resolve their relationships depend on the amount of polymorphism relative to the extent of divergence. Thus, it would be appropriate to use small samples per groups when most of the variation occurs among groups [62]. However, in such cases other inferences should be taken with caution as some estimates, for example those based on genetic diversity, could be biased. Sampling at each site was conducted during the same field season. Total genomic DNA was extracted according to standard CTAB and phenol-chloroform extraction procedures [63].

In order to compare the genetic structure between mitochondrial and nuclear markers, we used a microsatellite data set from a previous study about the effects of human impacts on the genetic variability of *Z. quitzeoensis *[49]. However, not all the populations included here for the mtDNA study were available at that time, and therefore the number of analysed populations differs between marker types.

### mtDNA sequencing and phylogenetic analysis

Two overlapping fragments of the cytochrome *b *gene (1140 bp total) were amplified via polymerase chain reaction (PCR) from 80 specimens distributed in 12 sampling sites. The primers used were those used in Machordom & Doadrio [64]. The amplification process was conducted using the conditions described elsewhere [21]. PCR products were sequenced in an ABI PRISM 3700 DNA analyser. Chromatograms and alignments were visually checked and verified. All sequences were deposited in GenBank under accession numbers: EU679420-EU679499.

We used phylogenetic tree-building algorithms to infer the phylogenetic relationships among sequence haplotypes. Maximum Parsimony Analysis (MP) was performed by heuristic searching with the tree-bisection-reconnection (TBR) branch swapping algorithm and random stepwise sequence addition using 10 replicates. Two different weights were given to the characters; first all characters were equally weighted, and second, transversions (Tv) were assigned eight times the weight of transitions (Ti) according to the empirically determined Tv/Ti ratio for *Zoogoneticus *obtained in PAUP 4.0b10 [65]. The robustness of the MP topologies was assessed by bootstrapping with 1000 replicates (full heuristic search) of 10 random stepwise addition replicates each. The model of DNA substitution that best fitted the data set was selected using MODELTEST 3.7 [66] using the Bayesian information criterion (BIC) for each codon position and the Akaike information criterion (AIC). A Neighbour-Joining (NJ) phylogram was obtained using maximum-likelihood distances according to the model selected by AIC. Bootstrap values for this analysis were obtained from 1000 replications. All phylogenetic analyses were performed using PAUP 4.0b10. Bayesian analysis was conducted with MrBayes 3.1.1 [67]. By simulating a Markov Chain Monte Carlo reaction for 2 × 10^6 ^cycles and using the substitution model obtained for each codon position selected by BIC criterion, 20,000 trees were generated, 1000 of which were burned and discarded. Posterior clade probabilities were used to assess node support. To identify ancestral and derived haplotypes, the trees were rooted using *Zoogoneticus tequila*, the sister species of *Z. quitzeoensis*, as outgroup, and a molecular clock of 0.9% per million years was applied to the pairwise uncorrected *p *genetic distances [21].

Evidence of positive selection was sought using a codon-based approach as implemented in Datamonkey [68]. This method does not need to assume equal synonymous substitution rates throughout the sequence and allows to choose the most appropriate model for nucleotide substitution. We used the single likelihood ancestor counting (SLAC) and fixed effects likelihood (FEL) approaches [69] using a *P *value of 0.1. In both cases, ambiguities in the consensus sequence were averaged in the analysis.

### Nested Clade Analysis

We constructed a 95% statistical parsimonious un-rooted haplotype network using TCS 1.18 [70]. As a complementary method to those performed before, and considering the limitations and drawbacks of Nested Clade Analysis [48,71], we tested geographical association among haplotypes and clades, based on the most parsimonious haplotype network and followed by a nested cladistic analysis (NCA), as described by Templeton [34,72]. To test for significant associations between clades and geographical sites, nested contingency analysis [73] was conducted by the program GEODIS 2.2 [74]. The AUTOINFER 1.0 [75] software package was used to infer the most suitable population structure model and historical scenario for the observed geographical associations.

### Genetic structure based on mtDNA

Pairwise Φ_ST _values were calculated among all geographic populations as an estimate of genetic differentiation. To assess the significance of genetic differentiation at different hierarchical levels, an analysis of molecular variance (AMOVA) was performed as described by Excoffier *et al*. [76]. Populations were initially grouped according to previous biogeographical information [6]. In subsequent analyses, we considered the information obtained in both the phylogenetic and the NCA analyses, but other hierarchical arrangements were also tested. Statistical significance was assessed using 20,000 permutations. A Mantel test (100,000 permutations, [77]) served to evaluate correlations between linear geographic distances and genetic distances. All analyses were performed using ARLEQUIN 3.1. [78].

### mtDNA diversity and demographic history

Population genetic statistics, such as the number of polymorphic sites (S), haplotype diversity (*H*_*d*_, [79]), nucleotide diversity (π, [79]) and the average number of pairwise nucleotide differences (*k*, [80]) were calculated using DnaSP 4.0 [81].

To investigate the demographic history of the groups identified in the phylogenetic analyses and through the AMOVA results, a mismatch distribution analysis (MMD) of pairwise substitution differences among haplotypes was performed for the whole data set and the lineages obtained. Deviations from the constant population size model were further tested using the Harpending's raggedness index (*r*) [82]. To test for deviations from neutrality we used Tajima's *D *[53] and Fu's *F*s [83] tests as implemented in DnaSP 4.0 [81]. We used the MMD age expansion parameter (τ) to date the onset of population expansion [84]. This was done by calculating τ using the equation τ = *2μt*, were *μ *is the sum of per-nucleotide mutation rates in the DNA region under study (0.9% PMY; [21]) and *t *is time in generations (0.5 for goodeines).

### Microsatellite analysis

We examined a previous data set, consisting of 135 individuals of *Z. quitzeoensis *from 10 populations, genotyped for five microsatellite loci (Additional file 5) [49] to look for differences in the allele frequencies of the populations by estimating *F*_*ST *_between all sample pairs, according to Weir & Cockerham [85], using ARLEQUIN 3.1. The significance of these estimates was assessed using 10,000 data permutations corrected by Bonferroni adjustment [86].

Geographical and phylogenetical genetic variation were compared among populations and clades by AMOVA. We assessed genetic isolation by distance [87], testing for independence between *F*_*ST *_estimates and geographical distances using a Mantel test [77]; regression matrices of *F*_*ST*_/1-*F*_*ST *_values *versus *the linear distance between sample pairs. All these analyses are implemented in ARLEQUIN 3.1.

To determine relationships among the sampled populations a neighbour-joining tree was created using the POPTREE program [88] based on *D*_*A *_modified Cavalli-Sforza distances [89] with 5000 bootstrap replications.

Because of the uncertainty that the geographical assignment of individuals to populations could represent biologically significant entities, a Bayesian clustering method was conducted as implemented in the program STRUCTURE 2.1 [90]. We performed a series of independent runs from *K *= 1 to 8 populations assuming correlated allele frequencies and an admixture model. For each value of *K*, the MCMC scheme was run with a burn-in period of 5 × 10^5 ^steps and chain length of 5 × 10^6^. Multiple runs were performed for each *K *to assess convergence of the results. Mean log probabilities were used to calculate *ΔK *(i.e. a quantity based on the second-order rate of change of the log probability of data between successive *K *values), to find the true *K *following the method of Evanno *et al*. [35]. Global *F*_*ST *_values were calculated for each *K *to find out which of the structures inferred explained the highest percentage of genetic variation. To assess the level of admixture and gene flow between the central Mexican Basins, we ran a population assignment test using GeneClass 2.0 [91].

## Authors' contributions

OD–D conceived the study, collected the samples, participated obtaining the molecular data and analyses and drafted the manuscript. FA contributed in the data analyses and helped to draft the manuscript. GP–PdL conceived the study, participated in its design and coordination and helped to draft the manuscript. JLG–G participated obtaining the molecular data and analyses. ID conceived the study, participated in its design and coordination and helped to draft the manuscript. All authors read and approved the final manuscript.

## Supplementary Material

Additional file 1 Sampling sites and individuals analysed of *Zoogoneticus quitzeoensis*. The table provided summarizes the collection sites of *Zoogoneticus quitzeoensis*, their geographical coordinates, the number of individuals analysed for cytochrome *b *and microsatellites and Genebank accession numbers. Click here for file

Additional file 2 Substitution parameters and evolutionary models obtained for the different criterion and partitions of the cytochrome *b *data. The data provided summarizes the parameters of the evolutionary models obtained for the cytochrome b data after the AIC and BIC. Ti/Tv ratio, Empirical base frequencies, Gamma shape parameter and Proportion of invariant sites are provided. Click here for file

Additional file 3 Inferences for all clades showing a significant association in the nested clade analysis results provided in Figure 3. The table describes the chain of inference obtained for all the statitistically significant clades in the Nested Clade Analysis. Click here for file

Additional file 4 (Left) Mean Ln*P*(*X*|*D*) for each of the *K *populations inferred by STRUCTURE. (Middle) Number of *Zoogoneticus *populations with the highest posterior probability expressed as the *ΔK *(Evanno et al. 2005). (Right) Comparisons between Ln*P(D) *(black circles) and *F*_*ST *_values (white squares) obtained for the different *K *values inferred by STRUCTURE. This figure represents graphically the values of *LnP(X|D), ΔK *and *F*_*ST *_for each of the different genetic arrangements inferred by STRUCTURE. Click here for file

Additional file 5 Summary table of diversity and BOTTLENECK statistics for the microsatellite data of *Zoogoneticus quitzeoensis*. Modified from Domínguez-Domínguez et al. (2007). The table describes values of genetic diversity based on microsatellite data. Click here for file
