# Supplementary material for: Evolutionary history of the endangered fish Zoogoneticus quitzeoensis (Bean, 1898) (Cyprinodontiformes: Goodeidae) using a sequential approach to phylogeography based on mitochondrial and nuclear DNA data
Source: BMC Evol Biol. 2008 May 26;8:161. doi: 10.1186/1471-2148-8-161 (PMC2435552; doi:10.1186/1471-2148-8-161)
Supplement: Additional file 1 — Sampling sites and individuals analysed of Zoogoneticus quitzeoensis. The table provided summarizes the collection sites of Zoogoneticus quitzeoensis, their geographical coordinates, the number of individuals analysed for cytochrome b and microsatellites and Genebank accession numbers. [file 1471-2148-8-161-S1.pdf]

Additional file 1. Sampling sites with their corresponding geographical coordinates and number of *Zoogoneticus quitzeoensis* individuals analysed for cytochrome *b* and microsatellites

Basin	Site	*n*	Coordinates	GenBank Acc
Ameca River	Magdalena	6 / 7	20º 53' 29.4" N<br>104º 01' 55.2" W	
	Moloya	6 / 10	20° 54´ 4.4" N<br>104° 4´ 46.7´´ W	
	Veneros	9 / 0	20º71' 22.9" N<br>103º30' 29.9" W	
Chapala Lake	La Alberca	7 / 0	20º03'32.93" N<br>102º36' 33.1" W	
Lower Lerma River	La Platanera	5 / 12	19º55'15.72" N<br>102º15'04.91" W	
	La Luz	7 / 20	19º56'08" N<br>102º18'02.1 W	
	Orandino	6 / 10	19° 57' 21.8" N<br>102° 19' 29.7" W	
Middle Lerma River	San Francisco del Rincon	7 / 19	21º02' 47.3" N<br>101º50'9.3" W	
Cuitzeo Lake	Belisario	6 / 19	19º53'42.41" N<br>101º04'16.84" W	
	San Cristóbal	7 / 12	19º57'42" N<br>101º18'57" W	
	La Mintzita	6 / 17	19º38'40" N<br>101º16'28" W	
Zacapu Lake	Zacapu	9 / 9	19º49'35" N<br>101º47'10" W	

n=Number of specimens for mtDNA / microsatellites
