# Supplementary material for: Evolutionary history of the endangered fish Zoogoneticus quitzeoensis (Bean, 1898) (Cyprinodontiformes: Goodeidae) using a sequential approach to phylogeography based on mitochondrial and nuclear DNA data
Source: BMC Evol Biol. 2008 May 26;8:161. doi: 10.1186/1471-2148-8-161 (PMC2435552; doi:10.1186/1471-2148-8-161)
Supplement: Additional file 2 — Substitution parameters and evolutionary models obtained for the different criterion and partitions of the cytochrome b data. The data provided summarizes the parameters of the evolutionary models obtained for the cytochrome b data after the AIC and BIC. Ti/Tv ratio, Empirical base frequencies, Gamma shape parameter and Proportion of invariant sites are provided. [file 1471-2148-8-161-S2.pdf]

Additional file 2. Substitution parameters and evolutionary models obtained for the different criterion and partitions of the cytb data.

Criterion	Codon position	Model of evolution	Substitution model Ti/Tv ratio	Empirical base frequencies	Gama distribution shape parameter	Proportion of Invariables sites
**AIC**	All	TIM+G	---	A=0.2473<br>C=0.2717<br>G=0.1544<br>T=0.3266	0.5996	---
**BIC**	First	K80	10.31	Equal	---	---
	Second	HKY	7.73	A=0.2069<br>C=0.2695<br>G=0.1366<br>T=0.3870	---	---
	Third	TrN+I	---	A=0.3047<br>C=0.3063<br>G=0.0620<br>T=0.3270	---	0.5085
