# Supplementary material for: Evolutionary history of the endangered fish Zoogoneticus quitzeoensis (Bean, 1898) (Cyprinodontiformes: Goodeidae) using a sequential approach to phylogeography based on mitochondrial and nuclear DNA data
Source: BMC Evol Biol. 2008 May 26;8:161. doi: 10.1186/1471-2148-8-161 (PMC2435552; doi:10.1186/1471-2148-8-161)
Supplement: Additional file 3 — Inferences for all clades showing a significant association in the nested clade analysis results provided in Figure 3. The table describes the chain of inference obtained for all the statitistically significant clades in the Nested Clade Analysis. [file 1471-2148-8-161-S3.pdf]

Additional file 3. Inferences for all clades showing a significant association in the nested clade analysis results provided in Figure 3.

Clade number	Populations studied	Statistics	Chain of inference	Population inference
5.1	Lineage I	$\chi 2$=45.00 *P*=0.000	1-2-3-5-NO	Insufficient genetic resolution to discriminate between range expansion/colonization and restricted dispersal/gene flow.
3.5	La Luz – Orandino	$\chi 2$=12.00 *P*=0.001	1-2-3-5-6-13-YES	Long distance colonization possibly coupled to subsequent fragmentation followed by range expansion.
3.6	Ameca	$\chi 2$=18.41 *P*=0.011	1-2-3-5-6-13-14-NO-21	Long distance colonization and/or past fragmentation. Insufficient evidence to discriminate between long-distance movements of the organisms and the combined effects of gradual movements during past range expansion and fragmentation.
4.2	Chapala – Lower Lerma	$\chi 2$=45.65 *P*=0.000	1-2-3-5-6-13-YES	Long distance colonization possibly coupled to subsequent fragmentation followed by range expansion.
4.1	Lineage II	$\chi 2$=50.20 *P*=0.000	1-2-3-5-6-13-14-NO-21	Long distance colonization and/or past fragmentation. Insufficient evidence to discriminate between long-distance movements of the organisms and the combined effects of gradual movements during past range expansion and fragmentation.
Whole cladogram		$\chi 2$=80.00 *P*=0.000	1-19-NO	Allopatric fragmentation.
