# Supplementary material for: Evolutionary history of the endangered fish Zoogoneticus quitzeoensis (Bean, 1898) (Cyprinodontiformes: Goodeidae) using a sequential approach to phylogeography based on mitochondrial and nuclear DNA data
Source: BMC Evol Biol. 2008 May 26;8:161. doi: 10.1186/1471-2148-8-161 (PMC2435552; doi:10.1186/1471-2148-8-161)

Additional file 4.- (Left) Mean Ln*P*(*X*|*D*) for each of the *K* populations inferred by STRUCTURE. (Middle) Number of Zoogoneticus populations with the highest posterior probability expressed as the *ΔK* (Evanno et al. 2005). (Right) Comparisons between Ln*P(D)* (black circles) and F_{ST} values (white squares) obtained for the different *K* values inferred by STRUCTURE.

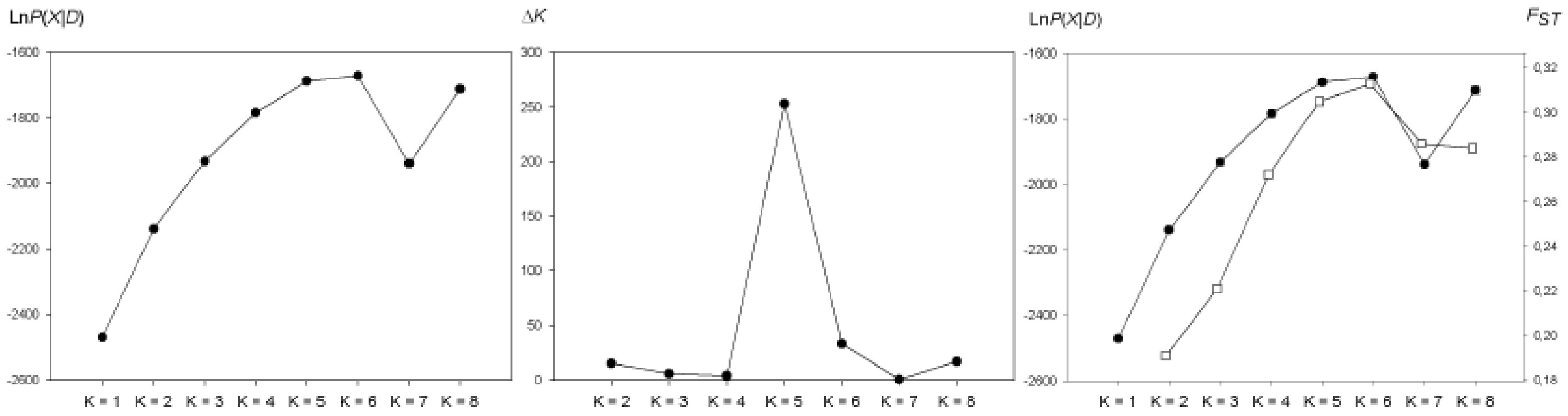

Supplement: Additional file 4 — (Left) Mean LnP(X|D) for each of the K populations inferred by STRUCTURE. (Middle) Number of Zoogoneticus populations with the highest posterior probability expressed as the ΔK (Evanno et al. 2005). (Right) Comparisons between LnP(D) (black circles) and FST values (white squares) obtained for the different K values inferred by STRUCTURE. This figure represents graphically the values of LnP(X|D), ΔK and FST for each of the different genetic arrangements inferred by STRUCTURE. [file 1471-2148-8-161-S4.pdf]
