# Supplementary material for: Evolutionary history of the endangered fish Zoogoneticus quitzeoensis (Bean, 1898) (Cyprinodontiformes: Goodeidae) using a sequential approach to phylogeography based on mitochondrial and nuclear DNA data
Source: BMC Evol Biol. 2008 May 26;8:161. doi: 10.1186/1471-2148-8-161 (PMC2435552; doi:10.1186/1471-2148-8-161)
Supplement: Additional file 5 — Summary table of diversity and BOTTLENECK statistics for the microsatellite data of Zoogoneticus quitzeoensis. Modified from Domínguez-Domínguez et al. (2007). The table describes values of genetic diversity based on microsatellite data. [file 1471-2148-8-161-S5.pdf]

Additional file 5.- Summary table of diversity and BOTTLENECK statistics for the microsatellite data of *Zoogoneticus quitzeoensis*. Modified from Domínguez-Domínguez et al. (2007).

Population	Biogeographic region	N	A	N_e	N_{PA}	H_O	H_E	F_{IS}	BOTTLENECK (IAM/SMM/TPM)
1.- El Moloya	Ameca River	10	5	3.69	4	0.61	0.6	-0.081	+/-/-
2.- Magdalena	Ameca River	7	4.2	3.09	2	0.6	0.65	-0.018	+/+/+
3.- Platanera	Lower Lerma	12	3.6	2.27	3	0.45	0.46	-0.185	+/-/+
4.- Orandino	Lower Lerma	10	6.2	4.17	9	0.58	0.63	0.085	-/-/-
5.- La Luz	Lower Lerma	9	4.6	2.29	8	0.45	0.49	0.163*	-/-/-
6.- San Francisco	Middle Lerma	19	3.8	2.4	3	0.38	0.42	0.093	-/-/-
7.- Zacapu	Zacapu Drainage	20	3	2.09	0	0.51	0.4	-0.235	+/+/+
8.- San Cristobala	Cuitzeo Drainage	12	6.2	4.17	1	0.5	0.46	0.145	+/+/+
9.- La Mintzita	Cuitzeo Drainage	17	4.8	3.05	1	0.48	0.47	-0.025	+/+/+
10.- Belisario	Cuitzeo Drainage	19	5.6	3	3	0.45	0.48	0.144	+/-/+

N: sample size; A: average number of alleles per locus; N_e: effective number of alleles; N_{PA}: number of private alleles; H_O: observed and H_E: expected heterozygosity (Nei 1973); F_{IS}: inbreeding index; *: significant departure from the Hardy-Weinberg Equilibrium ($p<0.001$); BOTTLENECK: results from the heterozygosity excess estimated by Wilcoxon sing-rank test ($P<0.05$), based on infinite allele model (IAM), stepwise mutation model (SMM) and two phase model (TPM).
